# Cariogenicity of *Streptococcus mutans* Glucan-Binding Protein Deletion Mutants

**Published:** 2013-12

**Authors:** David J Lynch, Suzanne M Michalek, Min Zhu, David Drake, Fang Qian, Jeffrey A Banas

**Affiliations:** 1 Dows Institute for Research, University of Iowa College of Dentistry, Iowa City, Iowa, USA; 2 Department of Microbiology, University of Alabama at Birmingham, Birmingham, Alabama, USA

**Keywords:** *S. mutans*, Caries, Biofilm, Glucan

## Abstract

*Streptococcus mutans* is a principal etiologic agent in the development of dental caries due to its exceptional aciduric and acidogenic properties, and its ability to adhere and accumulate in large numbers on tooth surfaces in the presence of sucrose. Sucrose-dependent adherence is mediated by glucans, polymers of glucose synthesized from sucrose by glucosyltransferase (Gtf) enzymes. *S. mutans* makes several proteins that have the property of binding glucans. We hypothesized that three of these glucan-binding proteins (Gbps), Gbps A, C and D, contribute to the cariogenicity of *S. mutans*. A specific pathogen-free rat model was used to compare the cariogenicity of *S. mutans* UA130 and a panel of mutants with individual or multiple *gbp* gene deletions. The mutants were also evaluated *in vitro* for properties related to cariogenicity, such as acidogenicity, aciduricity, and adhesion to glucan. Only a subset of Gbp mutants were attenuated for cariogenicity, with the combined loss of Gbps A and C most affecting smooth surface caries. The attenuation of Gbp mutant strains was unlikely due to differences in acid-related properties since the mutants were at least as acidogenic and acid-tolerant as the parental strain. Additionally, loss of Gbps did not reduce adhesion to a pre-formed biofilm of *S. sanguinis*. Analyses of the caries data with *in vitro* biofilm properties previously determined for the mutant panel found correlations between cariogenicity and biofilm depth and substratum coverage. It is concluded that Gbps contribute to the cariogenicity of *S. mutans* through a mechanism that may involve alteration of biofilm architecture.

## Introduction

Tooth decay is one of the most common infectious diseases affecting humans and is a significant health care issue in both modern and developing nations [1]. *Streptococcus mutans* is widely accepted as one of the most important etiologic agents associated with caries development and has been shown to directly cause caries in germ-free and specific pathogen-free rat models [2–4]. *S. mutans* possesses a variety of virulence factors that enable it to establish colonization, utilize a wide array of carbohydrate sources, produce acid and thrive at low pH. A trait that further separates this species from other oral plaque bacteria is the ability to accumulate in large numbers in the presence of dietary sucrose. This trait enables both adhesion to the tooth surface or to plaque colonies, and cohesion among dividing cells [5,6]. Sucrose is a substrate for three *S. mutans* enzymes, called glucosyltransferases (Gtfs), that cleave sucrose molecules and polymerize the glucose moieties into adherent glucans.

A search to find a glucan receptor on the cell surface resulted in the discovery of non-Gtf glucan-binding proteins [7,8]. The first of these glucan-binding proteins, GbpA, was isolated by Russell [9] using a dextran affinity column. Sequence analysis of the GbpA (then known as Gbp) revealed that the processed protein was a predicted 59 kD and contained a presumptive glucan-binding domain with repeats very similar to the Gtfs of *S. mutans* and other oral streptococci [10]. GbpB was isolated by Smith et al. [11], also by affinity chromatography, and was estimated to be 59 kD by SDS-PAGE. This protein is immunologically distinct from GbpA and was shown to be highly antigenic in humans and rodents. It should be noted that *gbpB* is an essential gene that is positively regulated by the VicRK system under stress and that the amount of extracellular GbpB was found to correlate with biofilm growth in a select group of clinical isolates [12]. GbpC was isolated by Sato et al. [13] from a mutant deficient in dextran-dependent aggregation (DDAG). GbpC most closely fits the definition of a cell receptor for glucan since it is a cell wall-anchored protein. Sequence analysis revealed that the GbpC shares some homology with the major streptococcal surface protein P1. Most recently, a fourth Gbp, GbpD, was discovered and isolated based on sequence analysis of the complete, annotated sequence of *S. mutans* UA159 strain [14]. GbpD possesses amino acid repeats similar to those in the glucan-binding domains of GbpA and the Gtfs. The GbpD was shown to have lipase activity and binds lipoteichoic acid of *S. sanguinis* [14].

Given the role of extracellular glucan in *S. mutans* sucrose-dependent adhesion, biofilm formation and cariogenicity, it is natural to speculate upon the roles of *S. mutans* proteins capable of binding glucan. Since the discovery of Gbps there have been several studies aimed at determining whether they play a role in *S. mutans* cariogenicity. Findings include decreased adhesiveness to glass [15–17], altered morphology of microcolony aggregates, weaker adhesion to nichrome wires [18] or even increased adherence to hydroxyl-apatite [19]. Collectively, these studies reveal that Gbps affect i*n vitro* properties potentially important for *in vivo* cariogenicity. *In vivo* analyses of the roles of Gbps, however, have been minimal and have yielded conflicting results [19,20]. The goal of this study was to systematically investigate the roles of Gbps in *S. mutans* cariogenicity.

## Materials and Methods

### 

#### Strains and culture media

The following strains were used in this study: *E. coli* JM109 (Promega, Madison, WI, USA); *S. mutans* UA130 (provided by Dr. Suzanne Michalek, University of Alabama-Birmingham) was used as the wild-type (WT) and parental strain for generation of Gbp mutants; and *S. sanguinis* ATCC 10556 (provided by Dr. David Drake, University of Iowa) was used in adhesion assays. The complete panel of Gbp mutants is listed in Table 1 and described in Lynch et al. [21]. Although GbpB also has an important role in biofilm formation, a *gbpB* mutation is lethal or results in abnormal growth [12,22] and so was not included among the Gbp mutant panel. *E. coli* was cultured in 2xYT broth (Becton, Dickenson and Co., Sparks, MD, USA) at 37 °C. *S. mutans* was cultured on Todd Hewitt (TH) (Becton, Dickenson and Co.) plates, in Chemically Defined Medium (CDM) (SAFC Biosciences, Inc., Lenexa, KA, USA), or in Tryptone-Yeast Extract-Glucose (TYG) medium and grown at 37 °C in an anaerobic chamber (5% CO_2_, 10% H_2_, 85% N_2_). Recovery of *S. mutans* from rats and from the *in vitro* adherence assay to a pre-formed *S. sanguinis* biofilm employed Mitis-salivarius-sucrose (MSS) agar and Mitis-salivarius-sorbitol-kanamycin-bacitracin (MSKB) agar, respectively.

#### *In vivo* caries study

*In vivo* experiments were performed in Fisher 344 rats determined to be specific pathogen-free (SPF) according to a previously published protocol [4]. Eight groups of five rats per cage (except one group with 7 rats per cage) were infected with the wild-type *S. mutans* UA130 or one of the Gbp mutants.

At 17 days of age, rat pups were weaned, removed from trexlar Isolators, and set up in groups of 5 rats per cage (with filter tops). Prior to infection (from 17 to 21 days of age) rats were provided antibiotic water (sulfamethoxazole-trimethoprim at 1 ml/47.3 ml H_2_O) and food (irradiated rat/mouse sterilizable diet 7917 from Harlan Laboratories, Madison, WI) soaked in antibiotic to reduce the oral commensal flora. Rats were taken off the antibiotic for 24 hours prior to infection with *S. mutans* (WT or one of the Gbp mutants), which was administered by oral swabbing each rat (daily from 22–25 days of age) with a fresh overnight culture of the appropriate strain. Rats were then provided diet MIT 305 (Harlan Teklad, Indianapolis, IN) and water containing 5% sucrose *ad libitum*. Oral swabs were taken 5 days post infection and plated on TH plates with appropriate antibiotic concentrations and incubated anaerobically at 37 °C to confirm colonization. On day 66 post infection, rats were sacrificed and the mandibles were removed and cleaned of excess tissue. The right mandible from each rat was placed in a tube containing 3 ml of phosphate buffer, which was placed on ice and sonicated to release bacteria from the teeth. Aliquots were diluted, plated on TH and MSS plates and incubated anaerobically at 37 °C to quantify the *S. mutans* present in the plaque. Right and left mandibles were then placed in 95% ethanol for 24 hours. The mandibles were then cleaned and stained overnight with murexide solution. After drying, the mandibles were scored for caries using the Keyes [23] method. Scores were recorded for the buccal, sulcal and proximal molar surfaces individually.

#### Glycolytic pH drop

*S. mutans* UA130 and mutants were grown in 15 ml of TH broth anaerobically overnight at 37°C. The OD_600_ was normalized to 1.0 and equal volumes of the cultures were pelleted by centrifugation at room temperature at 9000×G for 10 minutes. The rate of pH drop by planktonic suspensions of the bacterial strains in the presence of glucose was measured using a protocol based on that of Belli and Marquis [24]. Cells were rinsed twice by resuspension in 10 ml pre-warmed (37 °C) 50mM KCl, 1mM MgCl_2_ solution that was adjusted to pH 7 just prior to rinsing the cells. After pelleting cells following the second rinse, the cells were resuspended in 9 ml of the 50 mM KCl, 1mM MgCl_2_ solution and the pH adjusted to 7.2. After the pH stabilized, 1ml of 10 % glucose was added and the pH was measured and recorded every 30 seconds until it reached a plateau. A 50mM KCl, 1mM MgCl_2_ solution without bacteria served as a negative control for identical time points. Alternatively, when comparing the glycolytic pH drop between the parental (UA130) strain and the *gbpACD* triple mutant, a 2mM NaPO_4_ (pH 7.2) was used to rinse cells and 2 mM NaPO_4_ (pH 7.6 adjusted to pH 7.2) with 1% glucose was used to monitor the pH drop, allowing a slower overall pH drop.

#### Planktonic acid tolerance

A procedure adapted from Ma et al. [25] was used to assess acid tolerance of organisms grown in planktonic cultures. Cultures of *S. mutans* were grown overnight in 15 ml of TH broth at 37 °C anaerobically. These overnight cultures were diluted 1:2 and incubated at 37 °C until the OD_600_ reached 0.7. At this point, 2.5 ml of each sample culture was added to 6 ml each of 37 °C, pH 7.0 and pH 5.0 TYG broth. The TYG broth was made by adding 0.6% glucose to 2xYT (tryptone, yeast extract) and mixing 1:1 with 100 mM potassium phosphate buffer. For pH 7.0 TYG, the above was mixed with pH 7.0 phosphate buffer, whereas to make the pH 5.0 TYG, it was mixed with pH 4.5 phosphate buffer. The final concentration of the TYG was 0.3% glucose and 50 mM potassium phosphate.

Cultures were grown in TYG at their respective pH for one OD_600_ doubling. The cultures were then diluted 1:100 into 1% tryptone broth at pH 3.0 or pH 7.0 (control) and incubated for 2.5, 5 and 10 minutes. At the respective time points, aliquots were removed and diluted 1:100 in pH 7.4 PBS buffer. The diluted aliquots were plated on TH agar and incubated overnight anaerobically at 37°C. Acid tolerance data was expressed as a percentage of CFU from pH 3-incubated cultures relative to pH 7-incubated cultures.

#### Biofilm acid tolerance

*S. mutans* cultures were grown overnight in CDM to an OD_600_ of 1.0. 100 μl of the overnight cultures for the WT and each mutant were sub-cultured into 1.5 ml of CDM with 5% sucrose in a 24-well culture plate (Corning Inc., Corning, NY). Biofilms were grown overnight at 37°C in 5% CO_2_ with rotation (20 rpm) on a variable angle hematological rotator set at 30 degrees greater than horizontal. The biofilms were rinsed with PBS and 1 ml of 1% peptone (pH 7.0) was added to control wells for each strain. To four additional wells for each corresponding strain, 1 ml of 1% peptone (pH 3.0) was added and incubated for 0, 15, 30, and 45 minutes. At the specific time points, the wells were rinsed with PBS (pH 7.4) and then 1 ml of PBS was added to the wells. The wells were sonicated to disrupt the biofilms and then the sample was diluted and plated on TH plates. After 48 hours incubation in an anaerobic chamber at 37°C, the CFU were recorded. Biofilm acid tolerance data was expressed as a percentage of CFU from pH 3-incubated biofilms relative to pH 7-incubated biofilms.

#### Biofilm SDS tolerance

To measure the tolerance of biofilm bacteria to killing by SDS, overnight *S. mutans* biofilms were prepared in the same way as described for the experiments that measured biofilm acid tolerance. Biofilms were then washed twice with room temperature PBS (pH 7.4). One ml of PBS was added to control wells, and then experimental wells were incubated with PBS and either 0.01% SDS or 0.1% SDS for 10 minutes. The ten-minute time point was chosen because it was determined that substantial killing occurred after this amount of time at the two concentrations of SDS chosen. After 10 minutes, the PBS/SDS mixture was aspirated, the wells were rinsed 1x with PBS and then 1 ml of PBS was added to the wells. The wells were sonicated to disrupt the biofilms and then the samples were diluted and plated. CFU were counted after 2 days incubation in an anaerobic chamber at 37 °C and data were expressed as a percentage of CFU from SDS-incubated biofilms relative to PBS-incubated biofilms.

#### Adhesion to a *S. sanguinis* biofilm

As *S. mutans* is not considered a primary plaque colonizer, it was proposed that one role of Gbps could be to aid in adherence to glucan from an existing biofilm. To examine this possibility, we examined the initial adhesion events of *S. mutans* WT and Gbp mutant strains to a pre-formed *S. sanguinis* biofilm. *S. sanguinis* is a primary plaque colonizer. Flat bottom, 96-well culture plates (Corning Inc., Corning, NY) were used for adhesion experiments. Planktonic overnight cultures of *S. sanguinis* were grown in TY broth (3% tryptone, 0.06% yeast extract) anaerobically at 37°C. On day 2, plate wells were coated with 50 μg/ml BSA (Fisher Scientific, Pittsburgh, PA) in 20 mM NaHCO_3_ (Fisher Scientific, Pittsburgh, PA) for 1 hour at 37 °C. We found that *S. sanguinis* adhered to the BSA-coated plates better than to saliva-coated plates. While the wells were incubating in BSA solution, the overnight culture of *S. sanguinis* was diluted into 20 ml of pre-warmed 2xTY broth to an OD_600_ of 0.08.

This OD provided an inoculum of 1.0×10^6^ CFU that was critical in forming a stable *S. sanguinis* biofilm. 75 μl of a 4%-sucrose/2%-glucose solution (control wells had only 2% glucose) was added to sample wells and 75 μl of the *S. sanguinis* in 2xTY broth was added to each well (except the media-only control wells). The biofilm was grown without rotation overnight at 37°C in 5% CO_2_. On day 3, wells were aspirated and rinsed once with PBS and then incubated for 30 minutes with 0.1% BSA in PBS. Frozen stocks of test bacteria, previously prepared from log-phase cultures to ensure equivalent starting concentrations for each experiment, were thawed, diluted to working concentration (10^7^ bacteria) and added to the *S. sanguinis* biofilms along with 10,000 units/ml of catalase to prevent killing of the *S. mutans* by hydrogen peroxide generated by *S. sanguinis*. An aliquot of each stock sample was plated to determine the CFU of each inoculum. 50 μl of 0.2% BSA in PBS was added to each well followed by 50 μl of the specific sample bacterial preparation. The samples were incubated for 1, 2 and 3 hours at 37 °C in 5% CO2 with rotation (20 RPM). At each time point, samples were washed 5x with PBS and then incubated for 2 additional hours at 37 °C in 100 μl TY supplemented with 1% glucose. After incubation in the TYG, the biofilms were disrupted by vigorous pipetting and transferred to 900 μl of PBS. The samples were then sonicated to break up aggregates, diluted and plated on either TH plates for total biofilm counts (both species grew but S. sanguinus numerically overshadowed *S. mutans*) or MSKB plates selective for *S. mutans*.

### Statistical Analysis

Descriptive statistics of caries scores and the weights of the rats were computed, and one-way analysis of variance (ANOVA) with a Tukey post-hoc test was conducted to compare significant differences among the groups. Correlation between *in vivo* caries results and *in vitro* biofilm COMSTAT analyses were evaluated using the Pearson correlation test and Spearman rank correlation test, as appropriate. Results from acidogenicity experiments, acid tolerance and SDS tolerance experiments, and adhesion experiments were the averages of 3 independent trials and compared using one-way ANOVA with a Tukey post-hoc test. A p-value of less than 0.05 was used as a criterion for statistical significance, and statistical software SPSS was used for the data analysis.

## Results

### Cariogenicity

Examination of caries scores revealed that the *gbpACD* strain showed significantly reduced caries in both total number and severity (dentinal) (Table 2) across all surfaces of the tooth (Table 3) compared to the WT. The *gbpA*C strain also showed significant reductions in the total number and severity of carious lesions (Table 2), but this was limited to buccal (smooth) surfaces of the tooth (Table 3). The *gbpD* mutant showed significant attenuation only of dentinal caries (Table 2), and this was limited to sulcal (fissure) surfaces (Table 3). The *in vivo* data revealed no significant reduction in CFU recovered from the specific pathogen-free (SPF) rats (Table 2), which would suggest that there were no deficiencies in the abilities of any of the mutants to colonize teeth and establish biofilms in the presence of an existing flora. In fact, the *gbpAD* strain showed a statistically significant increase in colonization, but did not exhibit a significant increase in caries. It is unlikely that any differences in caries were due to changes in colonization potential. When analyzed by correlation tests, there was no correlation between the colonization levels of the strains and caries (data not shown).

While neither the *gbpA* nor *gbpC* mutants differed statistically in cariogenicity compared to the WT, loss of GbpC combined with the loss of GbpA, or loss of all three Gbps resulted in attenuation on buccal surfaces (*gbpAC*) and all surfaces (*gbpACD*) relative to the WT (Table 3). The mutant missing both GbpA and GbpC was also attenuated compared to the *gbpA* and *gbpC* individual mutants on buccal and sulcal surfaces, with the exception of sulcal D_x_ caries. The *gbpD* mutant strain had significantly fewer caries than the *gbpC* mutant on both buccal and sulcal surfaces, but combining the loss of GbpC with the loss of GbpD did not result in significant attenuation from the WT on any surfaces (Table 3). One of the more unexpected results was that the *gbpD* mutant was significantly attenuated compared to the *gbpAD* mutant for total caries (Table 2).

#### Acidogenicity of WT and mutant strains

To examine whether differences in acidogenicity could explain the caries data, we determined whether or not the mutant strains were capable of producing acid at the same level as the WT. Essentially, it was a test of the metabolic rates of acid production by the mutant strains to see if the Gbp mutations had any adverse effects on the organisms’ ability to metabolize sugar. Our results indicate that each of the single mutantsreduced the pH of an unbuffered solution, in the presence of glucose, at statistically similar rates as the WT (UA130) strain (data not shown). We also compared the ability of the WT strain and the *gbpACD* triple mutant strain to reduce the pH of a slightly buffered solution in the presence of glucose. Both strains reduced the pH at a similar rate (data not shown).

Measurement of acidogenicity in biofilm cultures using a protocol analogous to that used for planktonic cultures was not feasible due to an inability to normalize the biofilms to ensure identical numbers of bacteria at the start of the experiment. This prevented measurement of the rate of glycolytic pH drop in biofilm cultures. However, *in vitro* biofilm cultures of WT and Gbp mutants had a similar terminal pH of 4.5.

#### Acid tolerance

Having confirmed that the WT and Gbp mutant strains all had the same potential for acidogenicity, we measured the acid tolerance of planktonic cultures to determine if the Gbp mutations had any effect on the ability of the bacteria to undergo an acid tolerance response. The results in Table 4 show that, not only were there no significant decreases in acid tolerance, the *gbpACD* mutant showed a significant increase in acid tolerance in unadapted culture conditions. This increase was seen at the earliest time point (2.5 minutes) but was not observed at later time points. Since reduced sensitivity to acid killing, albeit limited to an early time point, could be considered an advantage over the wild-type, it was determined that there was no diminished level of acid tolerance in the Gbp mutant strains that could account for attenuation of cariogenicity. However, earlier initiation of acid tolerance suggests the possibility that the most severely attenuated strain has a mechanism for early induction of the acid tolerance response.

We speculated that changes in biofilm architecture secondary to the loss of Gbps may affect acid diffusion. Therefore, we next investigated the acid tolerance of WT and Gbp mutant strains in biofilm cultures. Table 5 shows that there were no decreases in acid tolerance among any of the mutant biofilms when compared to the WT biofilm. As with planktonic cultures, acid tolerance differences found among some mutants were in the direction of improved acid tolerance. Statistically significant increases in survival were observed for the *gbpA* and *gbpACD* mutants at the first time point. Mutant survival rates matched that of the WT at later time points similar to the pattern observed for the *gbpACD* mutant in planktonic culture.

**Figure 1:**
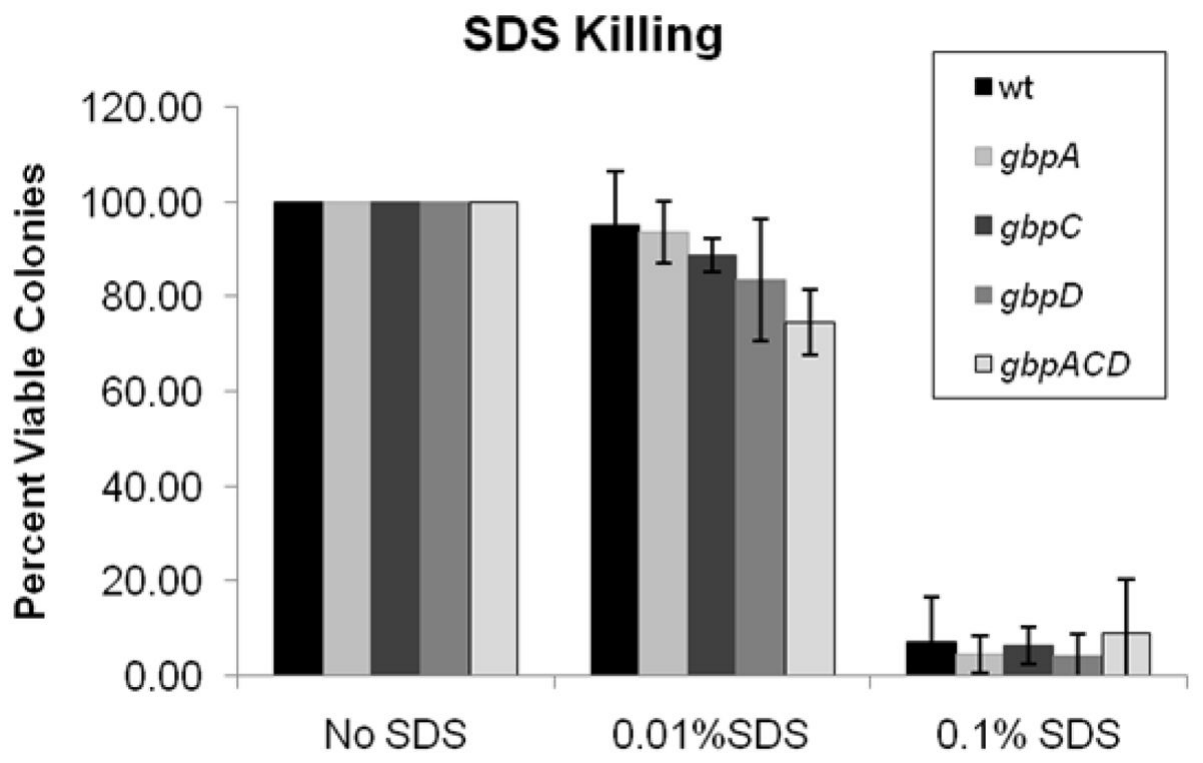
The effect of SDS on bacterial survival in biofilms. Shown are the percentages of surviving cells from overnight biofilms challenged with 0.1% or 0.01% SDS compared with recovered cells from unchallenged biofilms. Each value is the mean of 3 replicate biofilms. Error bars represent one standard deviation. No statistically significant differences were noted based on a one way ANOVA and Tukey post-hoc test.

The results of the acid killing experiments suggested that there is an earlier ATR or a higher basal level of ATR gene expression in at least some of the Gbp mutants. Although this was observed in both planktonic and biofilm cultures for the *gbpACD* strain, it was noted only in biofilm cultures for the *gbpA* mutant. Therefore, it was important to determine the extent to which changes in biofilm architecture may have altered acid diffusion and been responsible for early resistance to acid killing. In order to test this, another biofilm survival experiment was performed using sodium dodecyl sulfate (SDS) instead of acid. This eliminated the ATR and growth phase as a variable. Figure 1 shows that there were no significant differences in SDS killing among the WT and the Gbp mutant strains. The SDS tolerance data would seem to eliminate hindrance of diffusion, secondary to changes in biofilm architecture, as an explanation for increases in acid tolerance at early time points. This does not discount the possibility that differences in localized pH exist within biofilms or that the distribution of high or low pH regions could vary among WT and Gbp mutant strains. However, if these differences affect cariogenicity, it is not due to a negative effect on acid tolerance.

#### Adhesion to *S. sanguinis* pre-formed biofilm

The colonization data from the SPF rat experiment was an end point recovery of bacteria and could not reveal potential differences in accumulation rates. We entertained the possibility of a role for Gbps in initial attachment and adhesion to a pre-formed biofilm. *S. mutans* is generally considered a late colonizer to the tooth surface and therefore would most likely colonize an existing biofilm. Glucan-producing oral streptococcal species, such as *S. sanguinis* or *S. gordonii*, are considered early colonizers of the tooth pellicle [26]. Although not as prolific glucan producers as *S. mutans*, the extracellular polymeric matrix (EPM) they synthesize may represent possible adhesion targets for *S. mutans*. We grew *S. sanguinis* biofilms in the presence of sucrose to create an existing glucan-rich biofilm to examine binding of *S. mutans* WT and Gbp mutants. The *gbpACD* mutant was examined because it lacked all *Gbps* and was the most seriously attenuated in the rat model. We predicted that if Gbps were critical for binding to glucan in a pre-existing biofilm, then the *gbpACD* would show the largest loss of binding ability. We also examined adhesion of the *gbpA* strain because this strain showed the highest degree of substratum coverage in *in vitro* biofilms [21] and also retained full cariogenicity *in vivo*. The results, however, revealed that there was no loss of binding for either the *gbpACD* or the *gbpA* mutants compared to the WT strain (data not shown).

## Discussion

The purpose of this study was to test the hypothesis that Gbps play a role in the cariogenicity of *S. mutans*. Our hypothesis was based on the work of several groups [19,20,27] that found that individually deleting GbpA or GbpC impacted *S. mutans* cariogenicity in rat models, or altered cariogenic properties such as adhesion to smooth surfaces. Interestingly, our results indicated that only three strains were significantly attenuated for cariogenicity in our SPF rat model – *gbpAC* and *gbpACD* strains for total caries and severe caries, and the *gbpD* strain for severe caries. We found no significant differences in the caries scores between the WT and *gbpA* strain. Hazlett et al. [19] using the same bacterial strain (UA130 *gbpA*::erm), observed a significant increase in *gbpA* caries compared to the WT in a germ-free rat model. In that study, the rats were sacrificed after a shorter time period (30 days, as opposed to 66 days in the SPF rat experiment). It is possible that since we saw a slight increase in overall caries scores in the *gbpA* strain, the longer timeframe of our experiment may have allowed the WT caries incidence to “catch up” to that measured for *gbpA*-infected rats. However, an alternative explanation is that the presence of an indigenous microflora mitigated the increased cariogenic effect of a *gbpA* biofilm.

Studies by both Nakano et al. [27] and Matsumura et al. [20] evaluated the cariogenicity of *gbpA* and *gbpC* mutants. Matsumura et al. created individual mutants for *gbpA* and *gbpC* using the MT8148 parent *S. mutans* strain. Caries scores in SPF rats showed a significant reduction in caries in both mutant strains compared to the parental strain, but no differences in smooth surface (buccal) caries among any of the strains. Based on earlier published protocols from this group, we assume that their rats were infected for 50 days with the *S. mutans* strains before sacrifice, making it difficult to directly compare our results. Interestingly, they recovered significantly fewer CFU of the *gbpC* mutant than the other two strains at 10 days, but observed no differences in recovery at later time points. This suggests that Gbps may affect the rate of caries progression, in addition to incidence, and therefore the duration of a cariogenicity study can affect the nature of the outcome. It is also possible that the genotype of the parental strain significantly influences the effect of knocking out Gbps. Nakano et al. used two blood isolates that had either a mutation in the *gbpC* gene or mutations in both *gbpA* and *gbpC* to examine cariogenicity in a SPF rat model using lab strain MT8148 as a WT control. They observed no differences in cariogenicity of their *gbpC* strain when compared to their WT, but the strain lacking both *gbpA* and *gbpC* was significantly attenuated. Our results are in agreement with those of Nakano et al. in that a *gbpC* mutation alone was not attenuated, but a *gbpC* mutation combined with a *gbpA* mutation resulted in a reduction in caries.

We observed that Gbps contribute to the ability of *S. mutans* to induce caries in the presence of sucrose. Of the individual Gbp mutants, only the *gbpD* strain was attenuated relative to the parental. We propose that the combined loss of Gbps A and C has the most dominant effect on *S. mutans* cariogenicity, though the additive loss of GbpD extends the magnitude and breadth of the attenuation. While the loss of GbpD alone mostly affects caries development on sulcal surfaces, the combined loss of Gbps A and C has its greatest impact on smooth (buccal) surfaces. Clearly, each Gbp makes a unique contribution to the caries process, but the mechanistic contributions of each cannot be explained by the *in vivo* results alone. We can only speculate why the *gbpD* mutant is attenuated but the *gbpAD* and *gbpCD* mutants are not. It would appear that the loss of either GbpA or GbpC in some way compensates for the loss of GbpD, but when paired, the *gbpAC* mutant is significantly less cariogenic.

In order to better understand the basis for how Gbps contribute to cariogenicity, the single knockout (representing each Gbp) and triple knockout (the most attenuated strain) mutants were chosen for examination of the caries-related phenotypes of acid production, acid tolerance, and adherence to a pre-formed biofilm. The results of acid challenge experiments showed that while there was a possibility that Gbp mutants initiate an acid tolerance response or stress response earlier than the WT strain, there is no diminishment of their ability to produce acid or tolerate acidic environments. These results partly differ from those of Matsumoto-Nakano et al. [28] who reported that a *gbpA* mutant, but not a *gbpC* mutant, was significantly more sensitive to an acid challenge than the parental. Matsumoto-Nakano et al. also reported that their *gbpA* and *gbpC* mutants were deficient in dextran binding, whereas our mutants did not show any reduction in binding to a pre-formed biofilm of *S. sanguinis* formed in the presence of sucrose to ensure the presence of dextran. The basis for these differences is uncertain. However, we have noted highly variable, strain-specific *in vitro* expression levels of *gbp* genes in clinical isolates of *S. mutans* (unpublished data). Consequently, it is possible that the relative contributions of the different Gbps vary from one strain to another. Although this may complicate the assignment of function and mechanism to individual Gbps, it should be noted that the results of our study are consistent with a body of literature that finds that Gbps collectively, if not individually, contribute to *S. mutans* cariogenicity.

It should also be noted that microarray analyses comparing gene expression in the full panel of Gbp mutants with that of the parental strain did not find any statistically significant differences, either for organisms grown planktonically or in a biofilm (unpublished results). For this reason and in the absence of phenotypic differences in acid-related properties, physical differences in the mutant biofilms were considered. For example, it was postulated that one possible explanation for attenuation of Gbp mutants would be that altered biofilm architecture would affect the rates of acid diffusion through the biofilm. The biofilm acid tolerance and SDS tolerance experiments showed no evidence of differences in the ability of acid or detergent to penetrate a WT or *gbp* mutant biofilm. However, it is also possible that a loss of biofilm cohesion in Gbp mutants prevented accumulation of bacteria in large structures that form concentrated zones of low pH that would promote caries development.

In order to further consider the idea that changes in biofilm architecture, secondary to loss of one or more Gbps, could be responsible for changes in caries, we took advantage of an opportunity to analyze potential correlations between the caries data from the current study and data obtained in an earlier study [21] that quantified *in vitro* biofilm properties of the same panel of Gbp mutants. The correlational analyses (Table 6) provided evidence that the percentage of substratum coverage is an architectural trait that is associated with cariogenicity. Intuitively, it would make sense that the presence of more metabolically active bacteria in contact with the tooth surface would be correlated with a higher caries rate. The average biofilm thickness was also correlated with caries scores. Biofilm thickness, when measured for *in vitro* biofilms formed in the presence of constant rotation, may be a good indicator of the cohesive properties of the bacteria within that biofilm. This is supported by the fact that no differences in biofilm architecture were evident between WT and *gbpA* strains when grown under stationary conditions [29] but were evident when grown with rotation [21]. Strains of *S. mutans* that show a greater level of cohesion in an in vitro biofilm may have the ability to form *in vivo* biofilms with taller microcolonies that perhaps confer a selective advantage in the mixed-species plaque environment on smooth, buccal surfaces. Correlational analyses revealed that the impact of a reduction in biofilm thickness was most pronounced for the buccal surfaces of teeth suggesting that the loss of Gbps has less of an effect on fissured or proximal surfaces that are more protected from salivary flow or disruptive mechanical forces. It is likely that mutation of one or more Gbps affected the ability to colonize and form large aggregates on smooth surfaces to a greater degree than on other surfaces of the tooth. This theory is supported by studies with Gtf mutant strains of *S. mutans* where buccal cariogenicity was affected to a greater degree than sulcal cariogenicity [30–32]. Overall, a loss of biofilm thickness may contribute to, but be insufficient for, reducing cariogenicity. Some mutants may have compensated for a loss of biofilm thickness by spreading across the substratum and increasing substratum coverage thereby maintaining WT levels of cariogenicity. The Gbp mutants attenuated for cariogenicity in our rat model were previously shown to have had numerically reduced levels of both biofilm thickness and substratum coverage compared to the parental strain [21].

There remains a possibility for a temporal relationship among the Gbps and biofilm formation. GbpC may induce dextran-dependent aggregation of *S. mutans* cells early in biofilm development but it may be GbpA and GbpD that maintain microcolony cohesiveness throughout further maturation of the biofilm. Evidence supporting regulated expression of GbpC was provided by Biswas et al. [33], where expression of *gbpC* mRNA was observed to peak at mid-log phase and was extremely diminished in stationary phase cultures.

In conclusion, the loss of certain Gbps, individually or in combination, significantly attenuates the cariogenicity of *S. mutans*, revealing unique functions for individual Gbps and the complexity of how they interact with each another. Attenuation is correlated more with cohesion-related biofilm properties than with the acid-related phenotypes of Gbp mutants.

## Figures and Tables

**Table 1:** *S. mutans* strains used in this study.

Strain	Mutation	Antibiotic Resistance	Reference
UA130	None; serotype c strain	None	Dr. S. Michalek; UAB
*gbpA*	Δ*gbpA*	Erm	[34]
*gbpC*	Δ*gbpC*	Spec	[21]
*gbpD*	Δ*gbpD*	Km	[21]
*gbpAC*	Δ*gbpA,* Δ*gbpC*	Erm, Spec	[21]
*gbpAD*	Δ*gbpA,* Δ*gbpD*	Erm, Km	[21]
*gbpCD*	Δ*gbpC,* Δ*gbpD*	Spec, Km	[21]
*gbpACD*	Δ*gbpA,* Δ*gbpC,* Δ*gbpD*	Erm, Spec, Km	[21]

Gbp genes were knocked out via allelic replacement individually and in combination. Unique antibiotic resistance cassettes were used to replace gene fragments of each gene allowing for multiple mutations within the same strain. Antibiotic abbreviations: Erm, erythromycin; Spec, spectinomycin; Km, kanamycin.

**Table 2:** Overall Cariogenicity of WT and Gbp Mutants.

Cariogenicity on all Surfaces								
	WT	*gbpA*	*gbpC*	*gbpD*	*gbpAC*	*gbpAD*	*gbpCD*	*gbpACD*
Total Caries (E)	51.4 ± 1.21	53.0 ± 2.51	58.2 ± 1.77	44.2 ± 4.12^c^	**40.8 ± 2.04**^a,c^	55.6 ± 2.42^d^	46.4 ± 0.87^c^	**33.9 ± 1.77**^a,c^
Dentinal Caries (D_x_)	22.5 ± 3.12	19.0 ± 2.43	24.8 ± 2.84	**8.2 ± 2.91**^c^	**8.6 ± 1.60**^c^	25.4 ± 5.13^d^	18.4 ± 0.75	**4.0 ± 0.98**^a,c^
Log CFU Recovered	4.06 ± 0.28	4.67 ± 0.28	3.89 ± 0.11	4.54 ± 0.09	4.91 ± 0.05	5.23 ± 0.23	3.21 ± 0.35	3.65 ± 0.13

The total scores of all enamel (E) lesions (top row) and excessive dentinal (Dx) lesions (middle row) across all tooth surfaces are shown. The bottom row shows the log value of the recovered *S. mutans* colonies that were counted on mitissalivarius/sucrose plates. Values represent the average caries scores of 5 animals per group (7 in *gbpACD*) plus or minus the standard deviation. Statistical analysis was done by ANOVA using a Tukey post-hoc test. Mutant values that are significantly different (p < 0.05) than the wild-type, within a given row, are shown in bold type. Also within a given row: superscript ‘a’ denotes mutant scores that are significantly lower than those for the *gbpA* strain; superscript ‘c’ denotes mutant caries scores that are significantly lower than those for the *gbpC* strain; and superscript ‘d’ denotes mutant caries scores that are significantly greater than the *gbpD* strain.

**Table 3:** Cariogenicity of WT and Gbp Mutants by Surface.

Surface Localization of Caries								
	WT	*gbpA*	*gbpC*	*gbpD*	*gbpAC*	*gbpAD*	*gbpCD*	*gbpACD*
Buccal Enamel (E)	20.6 ± 0.93	21.0 ± 1.73	24.4 ± 1.47	16.0 ± 2.19^c^	**13.2 ± 1.39**^a,c^	21.4 ± 2.68	17.4 ± 0.60	**11.9 ± 0.60**^a,c^
Buccal Dentinal (D_x_)	13.0 ± 1.00	12.8 ± 1.77	17.0 ± 0.89	7.4 ± 2.54^c^	**3.6 ± 0.81**^a,c^	14.6 ± 3.33	9.6 ± 0.68	**1.71 ± 0.52**^a,c^
Sulcal Enamel (E)	23.0 ± 0.84	24.0 ± 0.89	25.8 ± 0.37	20.2 ± 1.93^c^	19.6 ± 0.75^a,c^	26.2 ^d^ ± 0.97	21.0 ± 0.32^c^	**18.4 ± 0.57**^a,c^
Sulcal Dentinal (D_x_)	9.8 ± 1.53	6.0 ± 1.76	7.8 ± 2.08	**0.8 ± 0.37**^c^	5.0 ± 0.95	10.8 ± 1.83^d^	8.8 ± 0.37^d^	**2.3 ± 0.68**
Proximal Enamel (E)	7.8 ± 0.20	8.0 ± 0.00	8.0 ± 0.00	8.0 ± 0.00	8.0 ± 0.00	8.0 ± 0.00	8.0 ± 0.00	**3.57 ± 0.87**^e^
Proximal Dentinal (D_x_)	0.0 ± 0.00	0.2 ± 0.20	0.0 ± 0.00	0.0 ± 0.00	0.0 ± 0.00	0.0 ± 0.00	0.0 ± 0.00	0.0 ± 0.00

Values represent the average caries scores of 5 animals per group (7 in *gbpACD*) plus or minus the standard deviation. Statistical analysis was done by ANOVA using a Tukey post-hoc test. Mutant values that are significantly different (p < 0.05) than the wild-type, within a given row, are shown in bold type. Also within a given row: superscript ‘a’ denotes mutant scores that are significantly lower than those for the *gbpA* strain; superscript ‘c’ denotes mutant caries scores that are significantly lower than those for the *gbpC* strain; superscript ‘d’ denotes mutant caries scores that are significantly greater than the *gbpD* strain; and superscript ‘e’ denotes mutant caries score that is significantly lower than scores from all other strains..

**Table 4:** Survival of Planktonic WT and Gbp Mutants Following Acid Challenge.

Planktonic Acid Challenge – Percent Survival				
Unadapted Cultures	Time = 0	Time = 2.5 minutes	Time = 5 minutes	Time = 10 minutes
**WT**	134.03 ± 28.50	3.96 ± 3.94	0.36 ± 0.38	0.02 ± 0.03
***gbpA***	107.68 ± 41.86	0.31 ± 0.38	0.09 ± 0.10	0.01 ± 0.02
***gbpC***	105.16 ± 25.82	0.41 ± 0.50	0.08 ± 0.03	3.34 ± 6.66
***gbpD***	100.23 ± 11.70	0.69 ± 0.55	0.17 ± 0.08	0.04 ± 0.03
***gbpACD***	103.27 ± 25.66	**34.85 ± 24.70**	4.27 ± 5.26	0.32 ± 0.53
**Acid-Adapted Cultures**				
**WT**	102.84 ± 26.16	88.12 ± 14.72	78.83 ± 20.98	56.24 ± 17.41
***gbpA***	109.56 ± 25.08	89.48 ± 15.85	77.40 ± 12.65	34.89 ± 23.86
***gbpC***	120.02 ± 30.48	92.51 ± 17.50	60.18 ± 43.54	26.24 ± 19.57
***gbpD***	129.53 ± 16.52	75.26 ± 13.46	70.66 ± 7.50	25.07 ± 12.07
***gbpACD***	127.81 ± 28.06	89.92 ± 8.20	75.78 ± 20.59	54.80 ± 28.68

Shown are the percentages of surviving cells in planktonic cultures after acid challenge compared with survival in unchallenged cultures. Values represent the average of three independent trials plus or minus the standard deviation. Only the survival of the unadapted *gbpACD* strain at 2.5 minutes (emboldened) was significantly different than the WT as determined by ANOVA using the Tukey post-hoc test.

**Table 5:** Survival of Biofilm WT and Gbp Mutants Following Acid Challenge.

Biofilm Acid Challenge – Percent Survival				
	Time = 0	Time = 15 minutes	Time = 30 minutes	Time = 45 minutes
**WT**	100.00 ± 0.00	42.59 ± 15.39	27.87 ± 14.76	19.99 ± 18.08
***gbpA***	100.00 ± 0.00	**72.56 ± 11.22**	35.50 ± 9.64	23.70 ± 15.17
***gbpC***	100.00 ± 0.00	69.79 ± 22.74	38.37 ± 23.25	21.26 ± 13.25
***gbpD***	100.00 ± 0.00	64.26 ± 12.01	44.90 ± 23.32	31.10 ± 17.00
***gbpACD***	100.00 ± 0.00	**68.33 ± 12.67**	28.46 ± 4.70	19.27 ± 7.75

Shown are the percentages of surviving cells in planktonic cultures after acid challenge compared with survival in unchallenged cultures. Values represent the average of three independent trials plus or minus the standard deviation. Bold values are significantly different that the WT for a given time point as determined by ANOVA using the Tukey post-hoc test.

**Table 6:** Correlation Coefficients for Biofilm Parameters.

	Biofilm Parameters		
	Substratum Coverage	Biomass	Average Thickness
Total Caries (E)	**0.714**	0.500	**0.719**
Total Caries (D_x_)	0.548	0.500	**0.707**
Buccal Caries (E)	**0.714**	0.500	**0.719**
Buccal Caries (D_x_)	0.619	0.524	**0.826**
Sulcal Caries (E)	0.690	0.571	0.695
Sulcal Caries (D_x_)	0.476	0.500	0.575

Correlation matrix showing correlation coefficients between biofilm parameters and caries scores. Statistically significant correlations are in bold type.
